# Benefits of heat-killed *Lactobacillus acidophilus* on growth performance, nutrient digestibility, antioxidant status, immunity, and cecal microbiota of rabbits

**DOI:** 10.3389/fvets.2024.1361908

**Published:** 2024-03-01

**Authors:** Miao Xia, Chong LI, Diange Wu, Fengyang Wu, Lingchang Kong, Zifan Jia, Wenxiao Han, Saijuan Chen, Wei Fang, Yajuan Liu, Baojiang Chen

**Affiliations:** ^1^College of Animal Science and Technology, Hebei Agricultural University, Baoding, China; ^2^Mountainous Area Research Institute of Hebei Province, Hebei Agricultural University, Baoding, China; ^3^Agricultural Technology Innovation Center in Mountainous Areas of Hebei Province, Baoding, China; ^4^Biosource Biotechnology Co., Ltd., Shenzhen, China

**Keywords:** heat-killed *Lactobacillus acidophilus*, rabbit, growth performance, antioxidant status, immune capacity, cecum microbiology

## Abstract

**Introduction:**

Heat-killed probiotics, as a type of inactivated beneficial microorganisms, possess an extended shelf life and broader adaptability compared to their live counterparts. This study aimed to investigate the impact of heat-killed *Lactobacillus acidophilus* (*L. acidophilus*, LA) - a deactivated probiotic on the growth performance, digestibility, antioxidant status, immunity and cecal microbiota of rabbits.

**Methods:**

Two hundred weaned Hyla rabbits were randomly allocated into five equal groups (CON, L_200_, L_400_, L_600_, and L_800_). Over a 28-day period, the rabbits were fed basal diets supplemented with 0, 200, 400, 600, and 800 mg/kg of heat-killed LA, respectively.

**Results:**

Results revealed a significant reduction in the feed-to-gain ratio (F/G) in the L_600_ and L_800_ groups (*p* < 0.05). Additionally, the L_800_ group exhibited significantly higher apparent digestibility of crude fiber (CF) and crude protein (CP) (*p* < 0.05). Regarding digestive enzyme activities, enhanced trypsin and fibrinase activities were observed in the L_600_ and L_800_ groups (*p* < 0.05). Concerning the regulation of the body’s antioxidant status, the L_800_ group demonstrated elevated levels of superoxide dismutase (SOD) and total antioxidant capacity (T-AOC) in both serum and ileal tissue (*p* < 0.05). In terms of immune capacity modulation, serum tumor necrosis factor-α (TNF-α) levels were significantly lower in the L_600_ and L_800_ groups (*p* < 0.05), while immunoglobulin A (IgA) and immunoglobulin M (IgM) levels were higher (*p* < 0.05). Additionally, the L_800_ group exhibited a substantial increase in secretory immunoglobulin A (SIgA) levels in the intestinal mucosa (*p* < 0.05). In comparison to the CON group, the L_800_ group exhibited a significant increase in the relative abundance of *Phascolarctobacterium* and *Alistipes* in the cecum (*p* < 0.05). *Phascolarctobacterium* demonstrated a positive correlation with SIgA (*p* < 0.05), IgM (*p* < 0.01), and Glutathione peroxidase (GSH-Px) (*p* < 0.05), while displaying a negative correlation with TNF-α levels (*p* < 0.05). Concurrently, Alistipes exhibited positive correlations with IgA (*p* < 0.05), IgM (*p* < 0.05), SIgA (*p* < 0.01), GSH-Px (*p* < 0.05), SOD (*p* < 0.05), and T-AOC (*p* < 0.01), and a negative correlation with TNF-α (*p* < 0.05).

**Discussion:**

In conclusion, the dietary incorporation of 600 mg/kg and 800 mg/kg of heat-killed LA positively influenced the growth performance, nutrient digestibility, antioxidant status, immune capacity and cecal microbiota of rabbits. This highlights the potential benefits of utilizing heat-killed probiotics in animal nutrition.

## 1 Introduction

Probiotics, defined as living microorganisms with proven health benefits upon proper administration, have been extensively studied for their capacity to enhance host immunity, optimize gut health, and improve overall animal growth performance (1, 2). Commonly employed probiotic strains encompass *Lactobacillus acidophilus*, *Lactobacillus plantarum*, *Bacillus subtilis*, and *Saccharomyces cerevisiae* (3–5). However, the widespread utilization of live probiotics has unveiled associated risks, including variable colonization patterns, instability of live bacteria, and potential strain transfer across the intestinal barrier (6). Consequently, concerns regarding these risks have led to an increased adoption of paraprobiotics, specifically inactivated probiotics (7). Various methods, including heat, high pressure, and ultraviolet light, have been explored for probiotic deactivation, with heat treatment often preferred. This choice is due to its effectiveness, cost-efficiency, and ability to preserve probiotic integrity, ensuring prolonged functionality and stability. Heat treatment stands as a widely adopted approach for preserving probiotic attributes across different contexts (8). Recent studies have substantiated that non-viable bacteria, notably inactivated probiotics, can confer health benefits akin to their living counterparts. These benefits encompass the modulation of immunity, pathogen suppression, and the enhancement of gut microbes to uphold overall animal health. Notably, these functional attributes of inactivated probiotics are attributed to their release of lipophosphatidic acid (LTA), peptidoglycan, and extracellular polysaccharide (EPS) components (9, 10). Additionally, the ease of storage and transportation of inactivated probiotics, compared to live bacteria, further underscores their practical advantages (7, 11, 12).

The weaning period poses a critical juncture for rabbits, characterized by an underdeveloped digestive tract and exposure to multiple stressors, including dietary transitions and relocation to new enclosures, potentially impacting their overall growth and developmental trajectories (13). Post-weaning, the abrupt cessation of breast milk antigen supply results in a rapid decline in intestinal immunity (14). Consequently, rabbits become more susceptible to environmental variations, eliciting stress responses that can manifest as diarrhea and, in severe cases, culminate in death (15).

Prior research has demonstrated the positive impact of dietary supplementation with alive LA on enhancing the dietary conversion rate, promoting growth performance, and augmenting beneficial intestinal flora in rabbits (4). Moreover, LA has been shown to fortify immunity, improve the integrity of the intestinal barrier, diminish the occurrence of diarrhea, and reduce mortality rates (4, 5, 16). Despite these established benefits, there remains a scarcity of literature addressing the utilization of heat-killed LA in rabbit production. This study aims to bridge this knowledge gap by investigating the repercussions of incorporating heat-killed LA into rabbit diets. The investigation also encompasses an in-depth analysis of its effects on growth performance, digestibility, digestive enzymes, antioxidant status, and immune capacity in rabbits. The results of this research will provide valuable scientific insights and provide a powerful reference for the rational application of heat-killed LA in rabbit production.

## 2 Materials and methods

### 2.1 Animals and management

A total of 200 healthy Hyla meat rabbits, uniform in weight at 35 days of age, were selected and randomly allocated into 5 groups, each comprising 8 replicates, with 5 rabbits in each replicate. Rabbits in the control group (CON) were exclusively fed a basal diet without any supplementation. In contrast, rabbits in the 4 treated groups, namely L_200_, L_400_, L_600_, and L_800_, received basal diets enriched with 200, 400, 600, and 800 mg/kg of heat-killed LA, respectively, for a duration of 28 days. The heat-killed LA was sourced from Biosource Biotechnology Co., Ltd. (Shenzhen, China) and contained 1 × 10^9^ cfu/g of LA in a dried product stored at room temperature (the main ingredients in this product include inactivated *Lactobacillus acidophilus* and silica). The formulation of the base diet adhered to the nutritional recommendations for rabbits as outlined by De Blas (17). The composition and nutritional levels of the base diet are detailed in Table 1. Both feeding and digestion experiments were carried out at the rabbit farm located within the Animal Husbandry Teaching Base of Hebei Agricultural University. Prior to the commencement of the experiments, meticulous cleaning and disinfection procedures were implemented for all rabbit cages. The feeding trial spanned a duration of 35 days, encompassing a pre-feeding phase of 7 days and a subsequent standard trial period of 28 days. Throughout the trial, all rabbits were afforded *ad libitum* access to both feed and water.

**Table 1:** Composition and nutrient levels of the basal diet (air-dry basis, %).

Ingredients	Content	Nutrient level^b^	Content
Yellow maize grain	12.00	Dry matter	89.23
Wheat bran	15.00	Digestible energy (MJ/kg)	9.83
Chaff	5.00	Crude protein	15.09
Soybean meal	13.00	Ether extract	2.34
Peanut vine	20.00	Crude fiber	16.02
Peanut shell	10.00	Neutral detergent fiber	31.77
Artemisia argyi powder	5.00	Acid detergent fiber	18.91
Wheat middlings	6.00	Calcium	1.29
Corn germ meal	10.00	Total phosphorus	0.63
Sodium chloride	0.30		
DL-methionine	0.10		
L-lysine	0.10		
Premix^a^	3.50		
Total	100.00		

a The premix provided the following per kg of diets: Cu (as copper sulfate) 20 mg, Fe (as ferric sulfate) 70 mg, Zn (as zinc sulfate) 70 mg, Se (as sodium sulphate) 0.25 mg, Mn (as manganese sulfate) 10 mg, Co 0.15 mg, I 0.2 mg, VB1 2 mg, VB2 6 mg, VB12 0.02 mg, Pyridoxine 2 mg, Pantothenic acid 50 mg, Nicotinic acid 50 mg, Choline 1,000 mg, Biotin 0.2 mg, VA 101000 IU, VD 900 IU, VE 50 mg, VK 2 mg.

b Digestible energy was a calculated value, while the others were measured values.

### 2.2 Growth performance

The composition and nutritional levels of the base diet are detailed in Table 1. Initial body weight (IBW) and final body weight (FBW) were recorded at the beginning and at the end of the experiment (70 days of age) by weighing on an empty stomach. Record the feeding amount and remaining feed amount of each group of rabbits, calculate the average daily feed intake (ADFI), average daily weight gain (ADG) and feed-to-weight ratio (F/G). ADFI, ADG and F/G were calculated as follows: ADFI = total feed intake/experimental days; ADG = (FBW − IBW)/experimental days; F/G = ADFI/ADG.

### 2.3 Nutrient digestibility

At the end of the experiment, a 7 days digestibility test was conducted. During the trial, feces from each replicate were collected, weighed, and frozen at −20°C to be prepared for chemical analysis. Crude protein (CP), crude fat (EE), crude fiber (CF), acid detergent fiber (ADF), and neutral detergent fiber (NDF) and other nutrients in feed and feces were determined using the Association of Official Analytical Chemists (AOAC) method (18). Calculation of the digestibility of rabbits according to the total manure collection method. The digestibility of nutrients was calculated according to the following formula:


$$\mathrm{Nutrient}\;\mathrm{digestibility}\%=\left[\left(t-f\right)/t\right]\times 100$$

where *t* is the nutrient intake [g] during collection and *f* is the amount of nutrients excreted in the feces [g].

### 2.4 Samples collection

Upon completion of the experimental period, rabbits underwent a 12 h fasting period. Subsequently, one rabbit was randomly chosen from each replicate, and a blood volume of 10 mL was collected from the marginal ear vein. Blood samples were collected in vacuum containers and subjected to centrifugation at 3,000 × g for 10 min at 4°C to isolate the serum. The serum samples were preserved at −20°C until further analysis. Following blood collection, rabbits were humanely euthanized using the pentobarbital method. Tissue samples from the jejunum, ileum, and cecum were obtained using meticulously sterilized surgical instruments. The jejunum, ileum, and cecal chyme were carefully stored in sterile lyophilized test tubes. The 5 cm jejunum and ileum segments were placed on an ice tray and the mucosa was gently scraped off with a slide (the mucosa volume was 3–3.5 mL). The scraped mucous membranes were stored in sterile lyophilized test tubes at −80°C for subsequent analysis.

### 2.5 Immune capacity

The quantification of IgA, IgG, IgM, IFN-γ, TNF-α, IL-4, and IL-6 levels was conducted using enzyme-linked immunosorbent assay (ELISA). Similarly, intestinal mucosal Secretory SIgA and MUC2 were also assessed via ELISA kits. All ELISA kits utilized in the study were procured from Beijing Borealis Technology Co (Beijing, China), and specific procedural guidelines outlined in the respective kit instructions were followed for accurate implementation.

### 2.6 Digestive enzyme activity and antioxidant status

The α-amylase, trypsin, lipase activity, and cellulase activity of intestinal contents, as well as the total antioxidant capacity (T-AOC), glutathione peroxidase (GSH-Px), superoxide dismutase (SOD) activities and malondialdehyde (MDA) content in serum, jejunal and ileal tissues were measured using ELISA kits (Beijing Borui Long Term Technology Co (Beijing, China)).

### 2.7 16S rRNA gene sequencing and analysis

The cecum contents, approximately 5 mL, were collected in sterile cryopreservation tubes and stored at −80°C. Total genomic DNA was extracted from cecal digesta using the OMEGA Soil DNA Kit (M5635-02; Omega Bio-Tek, Norcross, GA, United States) following the manufacturer’s instructions. The quantity and quality of the extracted DNA were assessed using a NanoDrop NC2000 spectrophotometer (Thermo Fisher Scientific, Waltham, MA, United States) and agarose gel electrophoresis, respectively. PCR amplification of the V3–V4 region of the bacterial 16S rRNA gene was performed using forward primer 338F (5′-ACTCCTACGGGAGGCAGCA-3′) and reverse primer 806R (5′-GGACTACHVGGGTWTCTAAT-3′). The amplification procedure consisted of: 98°C for 5 min, followed by 25 cycles of 98°C for 30 s, 53°C for 30 s, and 72°C for 45 s, with a final extension at 72°C for 5 min. The amplicons were purified with Vazyme VAHTSTM DNA Clean Beads (Vazyme, Nanjing, China) and quantified using the Quant-iT PicoGreen dsDNA Assay Kit (Invitrogen, Carlsbad, CA, United States). After individual quantification, the amplicons were pooled in equal amounts, and paired-end sequenced (2 × 250 bp) at Shanghai Personal Biotechnology Co., Ltd. (Shanghai, China) on the Illumina NovaSeq Platform using the NovaSeq 6,000 SP Reagent Kit (500 cycles). To estimate microbial diversity in individual samples, alpha diversity indices, such as the Chao1 richness estimator, Shannon diversity index, Simpson index, and Goods_coverage. Observed_species at the amplicon sequence variant (ASV) level were calculated using the ASV table in QIIME2. Principal coordinate analysis (PCOA) was performed based on Bray–Curtis and UniFrac distance metrics.

### 2.8 Date analysis

Statistical data analyses were performed using Excel 2020 and SPSS 26.0 software. One-way analysis of variance (ANOVA) was used to test for significant differences between groups of data, while Duncan’s method was used for multiple comparisons. *p*-value <0.05 indicated significant differences between groups.

## 3 Results

### 3.1 Growth performance

The supplementation of heat-killed LA in the diet did not yield statistically significant differences (*p* > 0.05) in FBW, ADF, and ADG among rabbits, as indicated in Table 2. Nevertheless, noteworthy reductions were observed in the Feed-to-Gain ratio (F/G) within the L_600_ and L_800_ groups, and these reductions were statistically significant (*p* < 0.05).

**Table 2:** Effect of heat-killed *Lactobacillus acidophilus* on the growth performance of rabbits.

Item	Groups					SEM	*p*-value
	CON	L_200_	L_400_	L_600_	L_800_		
IBW, g	1257.71	1247.10	1248.39	1249.52	1233.99	8.426	0.943
FBW, g	2274.07	2270.25	2295.53	2312.49	2287.48	9.917	0.689
ADG, g/d	36.30	36.54	37.40	37.96	37.62	0.264	0.209
ADFI, g/d	158.17	155.00	158.85	156.67	155.46	0.535	0.215
F/G	4.38^a^	4.30^a^	4.26^a^	4.15^b^	4.15^b^	0.028	0.027

IBW, initial body weight; FBW, final body weight; ADFI, average daily feed intake; ADG, average daily gain; F/G, ratio of feed and gain. Data are means for 8 replicates of 5 rabbits per replicate. SEM, total standard error of the means (*n* = 8). ^a,b^Means with different superscripts in the same row are significantly different (*p* < 0.05).

### 3.2 Nutrient digestibility

As presented in Table 3, the digestibility of CP exhibited a notable increase in the L_800_ group, achieving statistical significance (*p* < 0.05). Moreover, the digestibility of CF and NDF in the L_600_ and L_800_ groups demonstrated a significant improvement when compared to the CON group (*p* < 0.05). Conversely, the digestibility of GE and other nutrients, including DM, ADF, EE, Ash, Ca, and P, did not manifest significant differences in rabbits when compared to the CON group (*p* > 0.05).

**Table 3:** Effect of heat-killed *Lactobacillus acidophilus* on apparent digestibility (%) of energy and nutrients in rabbits.

Item	Groups					SEM	*p*-value
	CON	L_200_	L_400_	L_600_	L_800_		
DM	52.12	51.39	52.71	50.14	51.03	0.550	0.646
GE	57.26	57.46	58.05	54.16	57.36	0.543	0.166
CP	76.70^b^	76.60^b^	76.67^b^	77.27^b^	79.99^a^	0.373	0.010
CF	19.43^c^	18.70^c^	20.90^bc^	26.04^ab^	29.91^a^	1.059	0.001
NDF	29.56^c^	30.80^bc^	29.34^c^	34.84^ab^	36.40^a^	0.781	0.004
ADF	20.41	20.96	25.00	26.68	25.59	1.256	0.403
EE	87.07	87.11	87.94	88.03	88.33	0.194	0.095
Ash	35.49	29.13	40.67	34.12	33.92	1.246	0.057
Ca	56.25	65.43	60.88	55.38	61.21	1.266	0.070
P	16.55	13.65	17.62	18.24	17.74	0.918	0.539

DM, dry matter; GE, gross energy; CP, crude protein; EE, ether extract; CF, crude fiber; NDF, neutral detergent fiber; ADF, acid detergent fiber; Ca, calcium; P, total phosphorus. SEM, total standard error of the means (*n* = 8). ^a,b,c^Means with different superscripts in the same row are significantly different (*p* < 0.05).

### 3.3 Digestive enzyme activity

**Figure 1:**
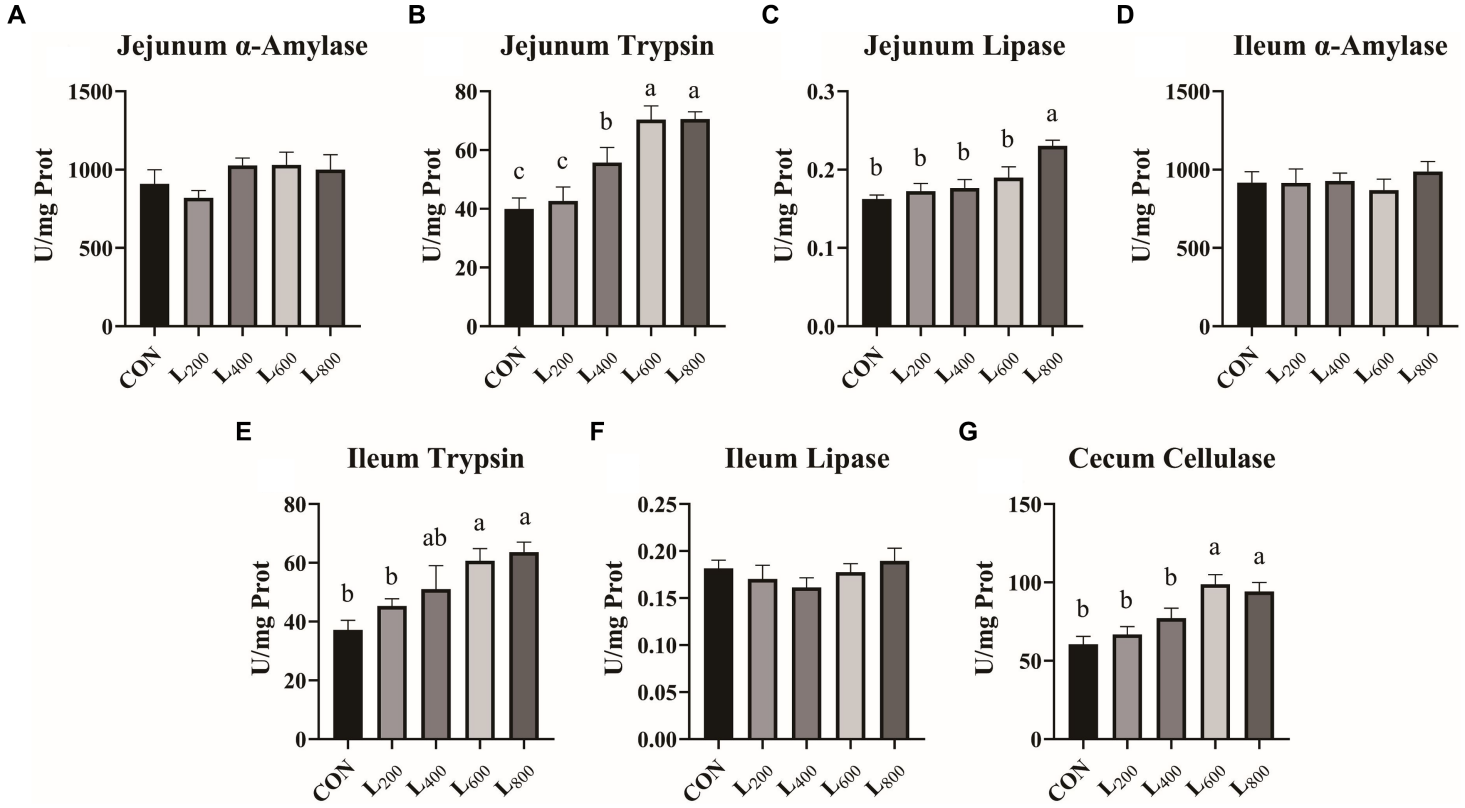
Effect of heat-killed *Lactobacillus acidophilus* on intestinal digestive enzymes in rabbits. Values are means ± standard error (*n* = 8). **(A–C)** α-Amylase, trypsin, lipase in jejunum; **(D–F)** α-Amylase, trypsin, lipase in ileum; **(G)** Cellulase in cecum. ^a,b,c^Means among the experimental groups with different letters are significantly different (*p* < 0.05).

The effects of the different treatments on intestinal digestive enzyme levels are presented in Figure 1. Compared with CON group, the activities of jejunum and ileum trypsin and cecum cellulase in L_600_ and L_800_ groups were significantly increased (*p* < 0.05) (Figures 1B,E,G), and the jejunum lipase activity in L_800_ group was also significantly higher (*p* < 0.05) (Figure 1C). Compared with CON group, α-amylase activity in jejunum and ileum and lipase in ileum of rabbits in experimental groups were not significantly different (*p* > 0.05).

### 3.4 Antioxidant indexes

**Figure 2:**
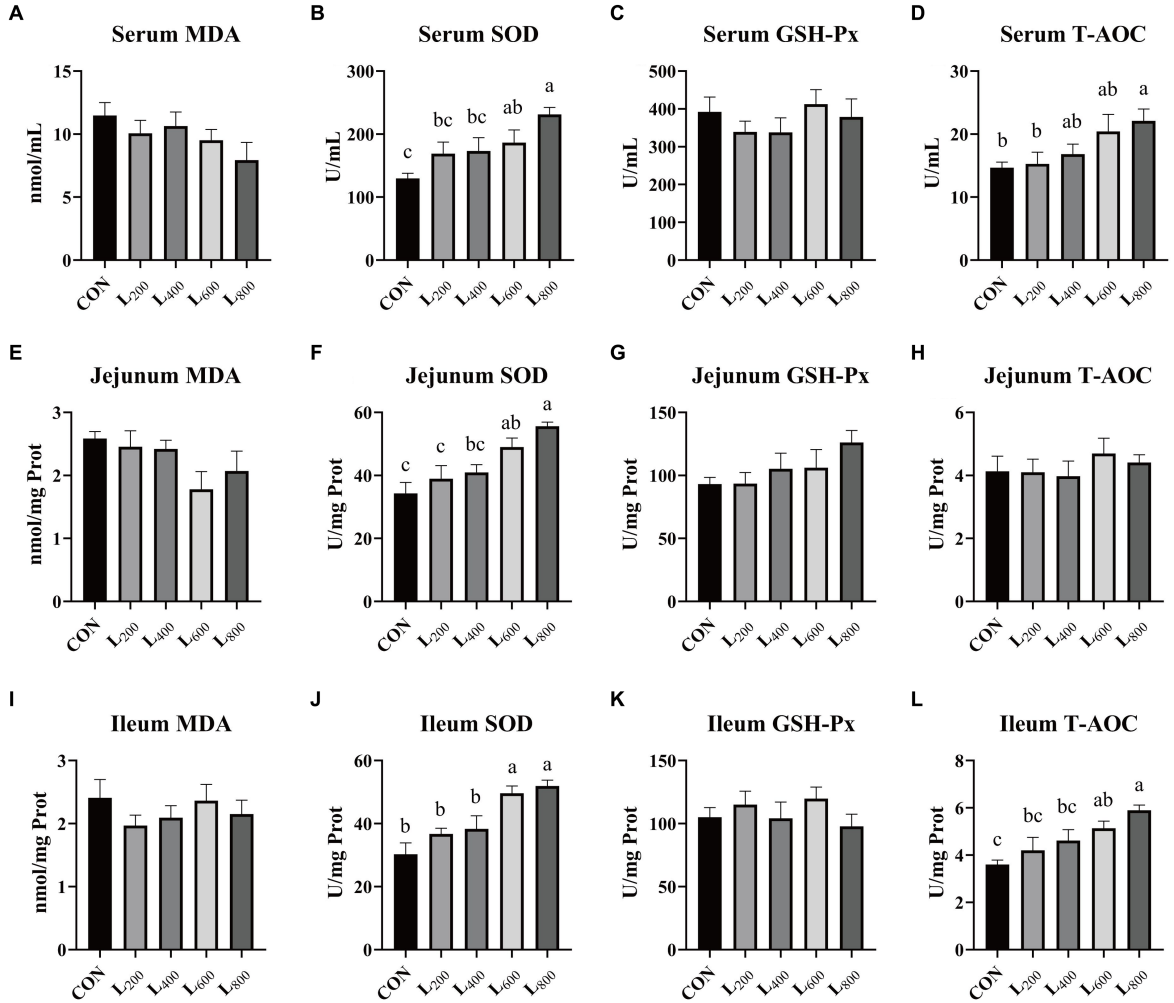
Effect of heat-killed *Lactobacillus acidophilus* on antioxidant status of rabbits. Values are means ± standard error (*n* = 8). **(A–D)** MDA, SOD, GSH-Px, TAOC in serum; **(E–H)** MDA, SOD, GSH-Px, TAOC in jejunum; **(I–L)** MDA, SOD, GSH-Px, TAOC in ileum. MDA, malondialdehyde; SOD, superoxide dismutase; GSH-Px, glutathione peroxidase; T-AOC, total antioxidant capacity. ^a,b,c^Means among the experimental groups with different letters are significantly different (*p* < 0.05).

In comparison to the CON group, the SOD activity in the serum and intestine tissue of the L_600_ and L_800_ groups exhibited a significant increase (*p* < 0.05) (Figures 2B,F,J). Additionally, the serum T-AOC activity was significantly elevated in the L_800_ group (*p* < 0.05, Figure 2D). Furthermore, the T-AOC activity in the ileum of rabbits in the L_600_ and L_800_ groups demonstrated a noteworthy increase in comparison to the CON group (*p* < 0.05) (Figure 2L). However, there were no statistically significant differences observed in MDA content and GSH-Px activity within the serum, jejunum, and ileum between the experimental groups and the CON group (*p* > 0.05).

### 3.5 Serum immune capacity

**Figure 3:**
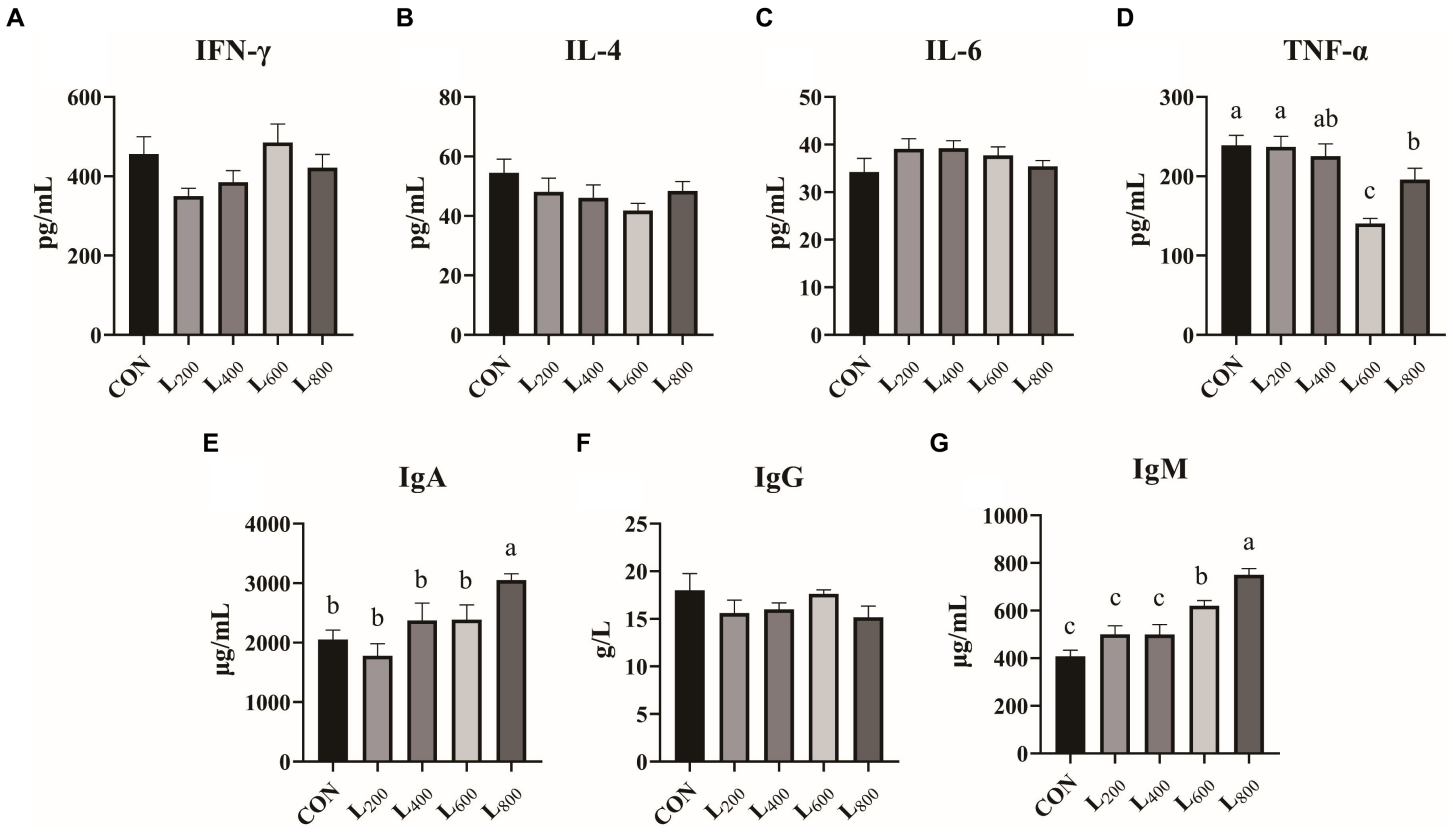
Effect of heat-killed *Lactobacillus acidophilus* on serum immunity in rabbits. Values are means ± standard error (*n* = 8). **(A–G)** serum immunological index. IFN-γ, interferon-γ; IL-4, interleukin-4; IL-6, interleukin-6; TNF-α, tumor necrosis factor-α; IgA, immunoglobulin A; IgG, immunoglobulin G; IgM, immunoglobulin M. ^a,b,c^Means among the experimental groups with different letters are significantly different (*p* < 0.05).

In Figure 3, it was shown that serum TNF-α levels were significantly reduced in the L_600_ and L_800_ groups compared with the CON group (*p* < 0.05) (Figure 3D). The serum IgA levels in the L_800_ group were significantly increased (*p* < 0.05) (Figure 3E). In addition, serum IgM levels were significantly higher in the L_600_ and L_800_ groups compared with the CON group (*p* < 0.05) (Figure 3G). And IFN-γ, IL-4, IL-6, and IgG levels were not significantly different compared with the CON group (*p* > 0.05).

### 3.6 Intestinal mucosal immune capacity

**Figure 4:**
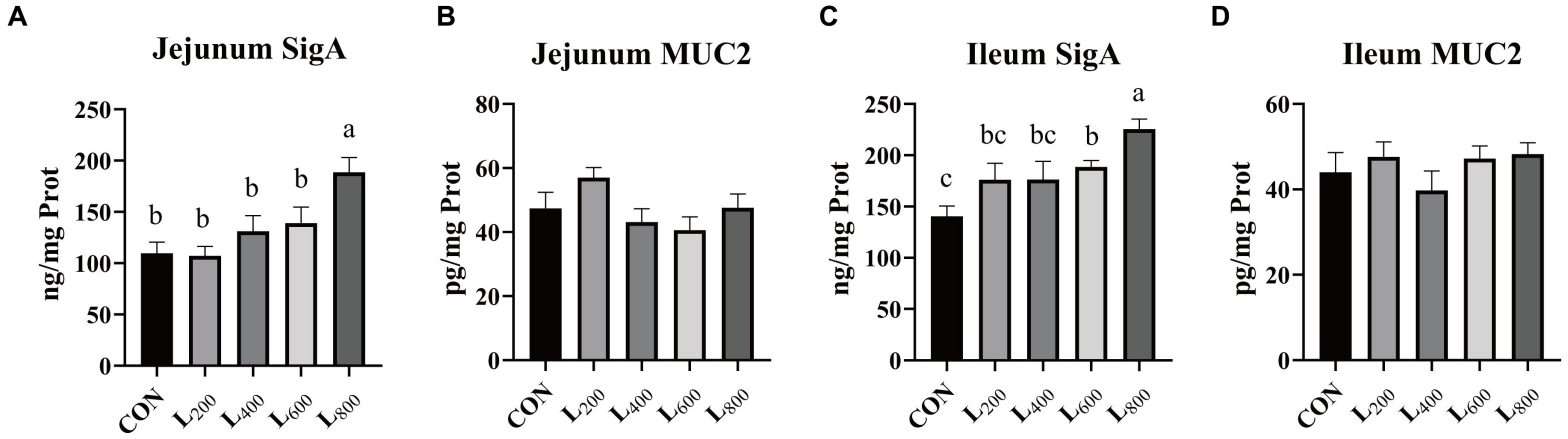
Effect of heat-killed *Lactobacillus acidophilus* on intestinal mucosal immunity in rabbits. Values are means ± standard error (*n* = 8). **(A,B)** SigA, Muc2 in jejunum; **(C,D)** SigA, Muc2 in ileum. SigA, Secretory immunoglobulin A; Muc2, Mucin 2. ^a,b,c^Means among the experimental groups with different letters are significantly different (*p* < 0.05).

The findings presented in Figure 4 indicate a substantial increase in SigA content in the jejunal mucosa of rabbits within the L_800_ group compared to the CON group (*p* < 0.05) (Figure 4A). Similarly, ileum mucosa was significantly increased in the L_600_ and L_800_ groups (Figure 4C). However, there were no significant differences observed in mucosal MUC2 content in the jejunum and ileum between the experimental groups and the CON group (*p* > 0.05).

### 3.7 Cecum microflora

**Figure 5:**
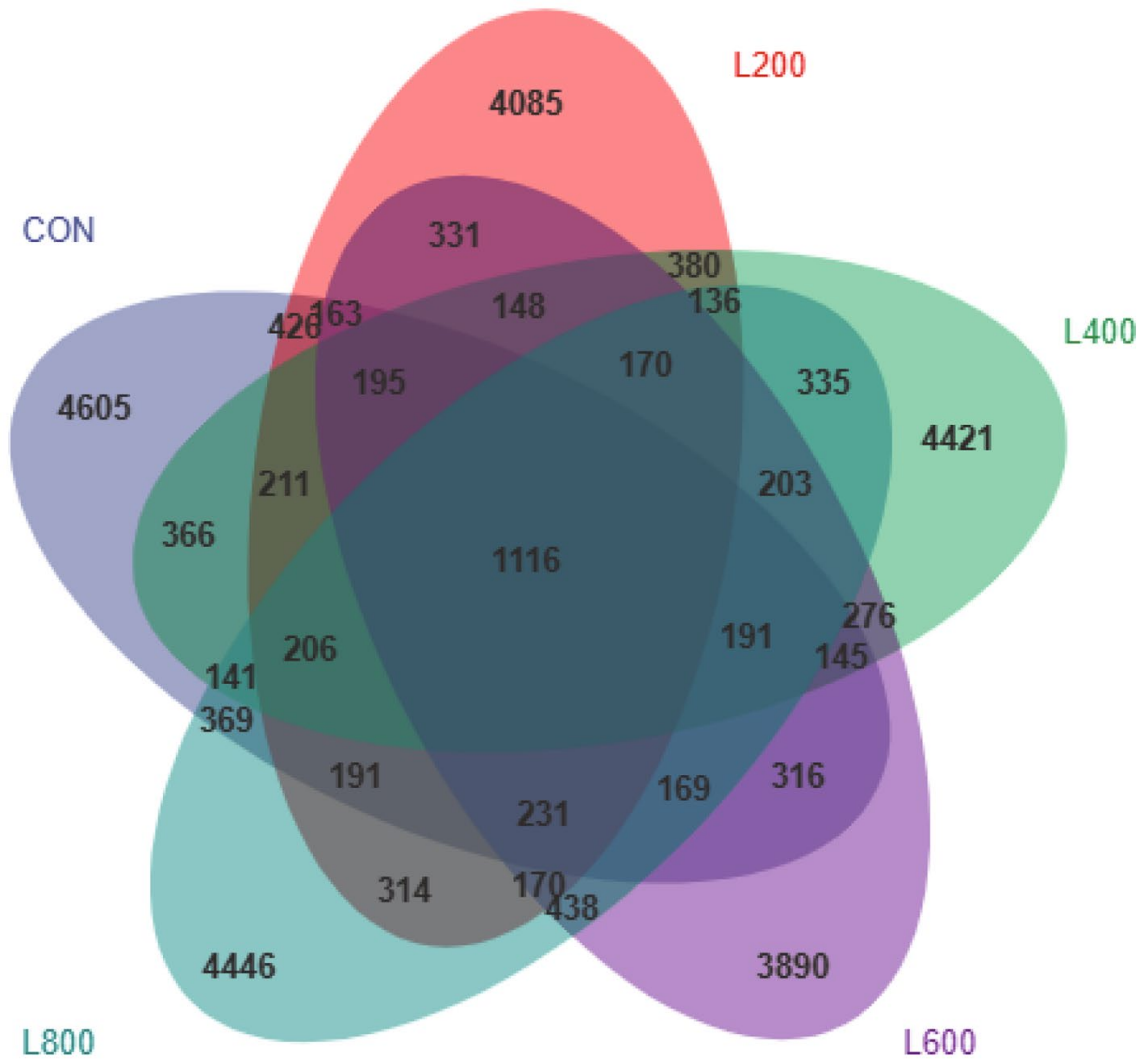
Venn diagram showing common and unique operational taxonomic units (OTUs) in the cecal digest of rabbits. According to the feature abundance table, the number of features in each group was calculated, and the number of common and unique features in each group was visualized by Venn diagram, if the feature existed in only one group, it was unique to that group.

**Figure 6:**
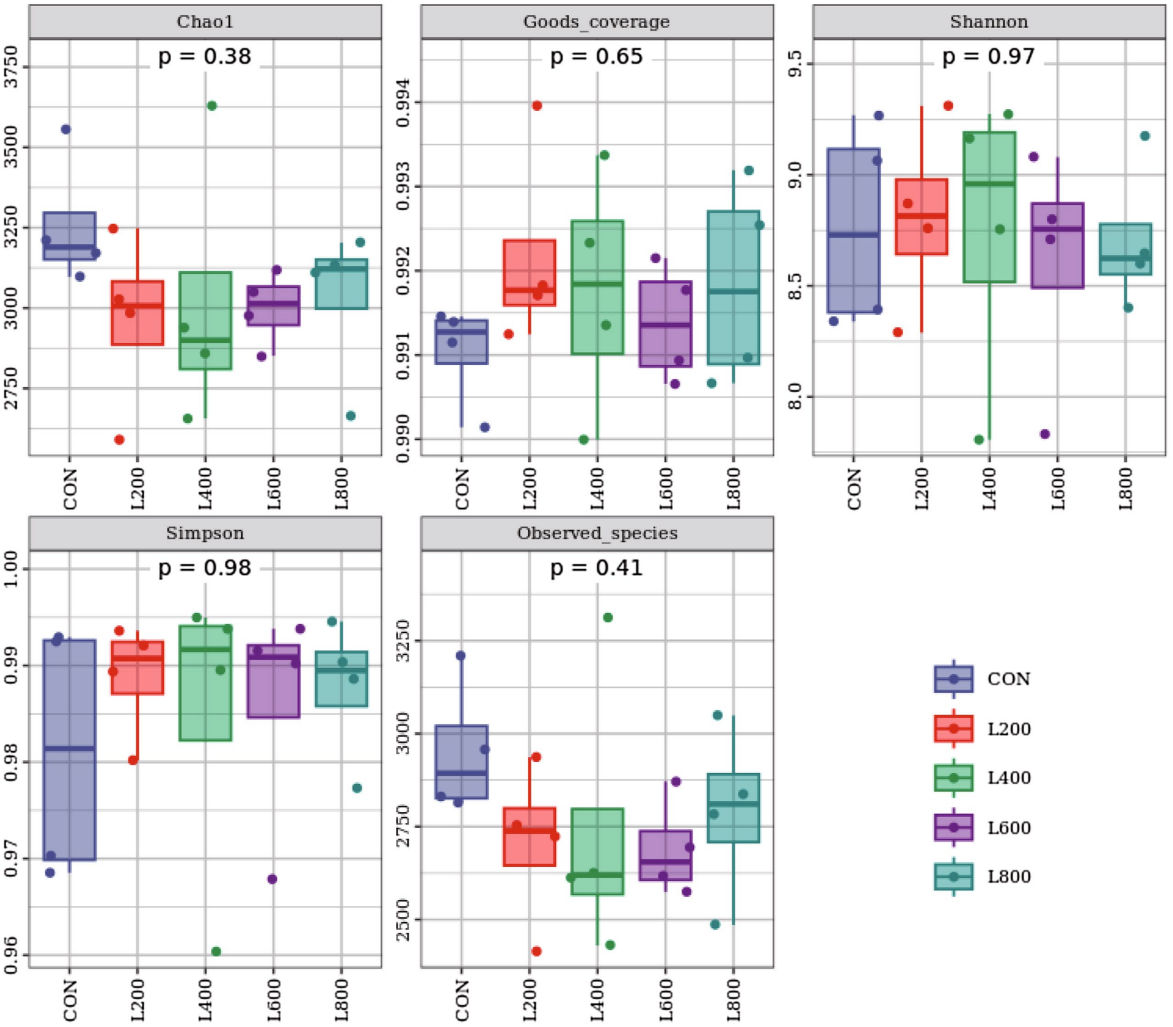
Alpha diversity indices of cecum microbiota. Chao 1 and Observed_otus indices mainly reflect the richness of the species in the sample; Goods_coverage reflects the low abundance feature coverage of the sample; Simpson and Shannon reflect the richness and evenness of the species together.

**Figure 7:**
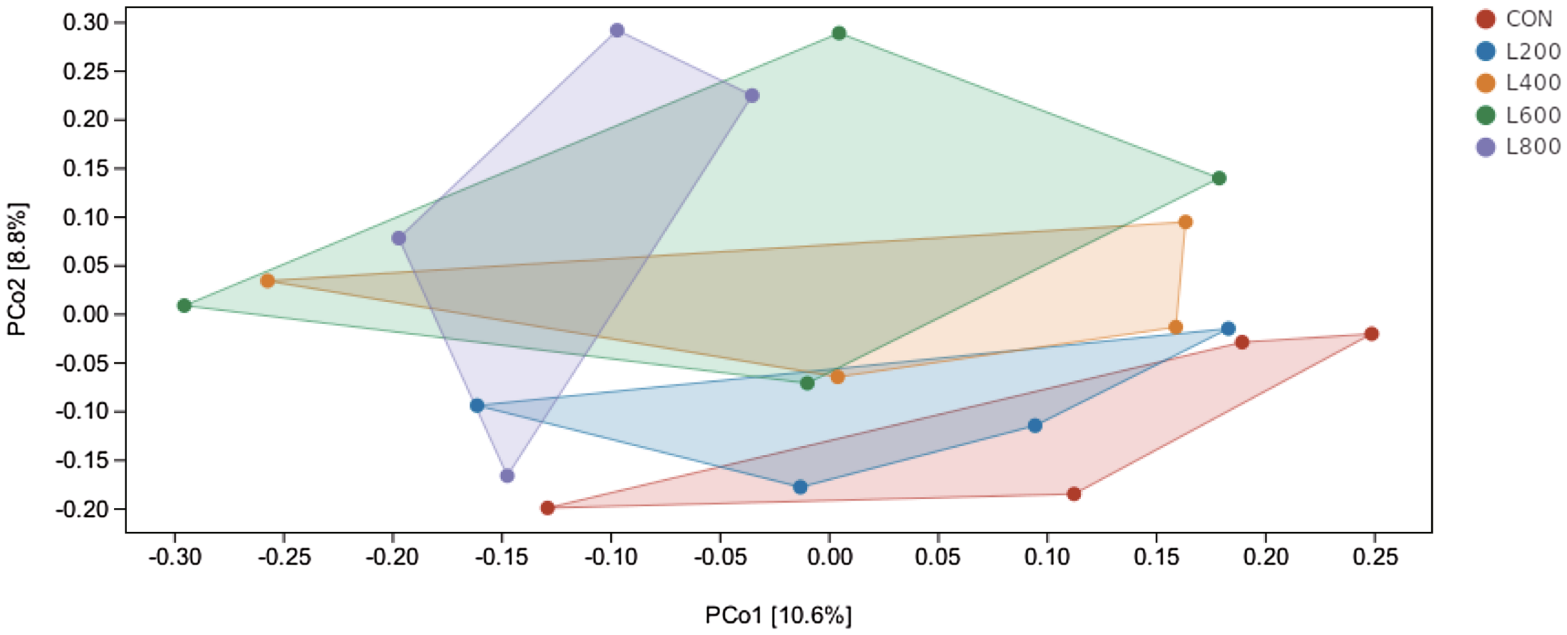
Principal co-ordinates analysis diagram of cecum microflora composition. Principal Coordinate Analysis (PCoA) is based on a sample distance matrix for ranking. The PCoA analysis allows the difference among the groups to be observed. The different colours in the results represent the different groupings and the closer the distance, the more similar the structure of the microbial composition and the less difference.

**Figure 8:**
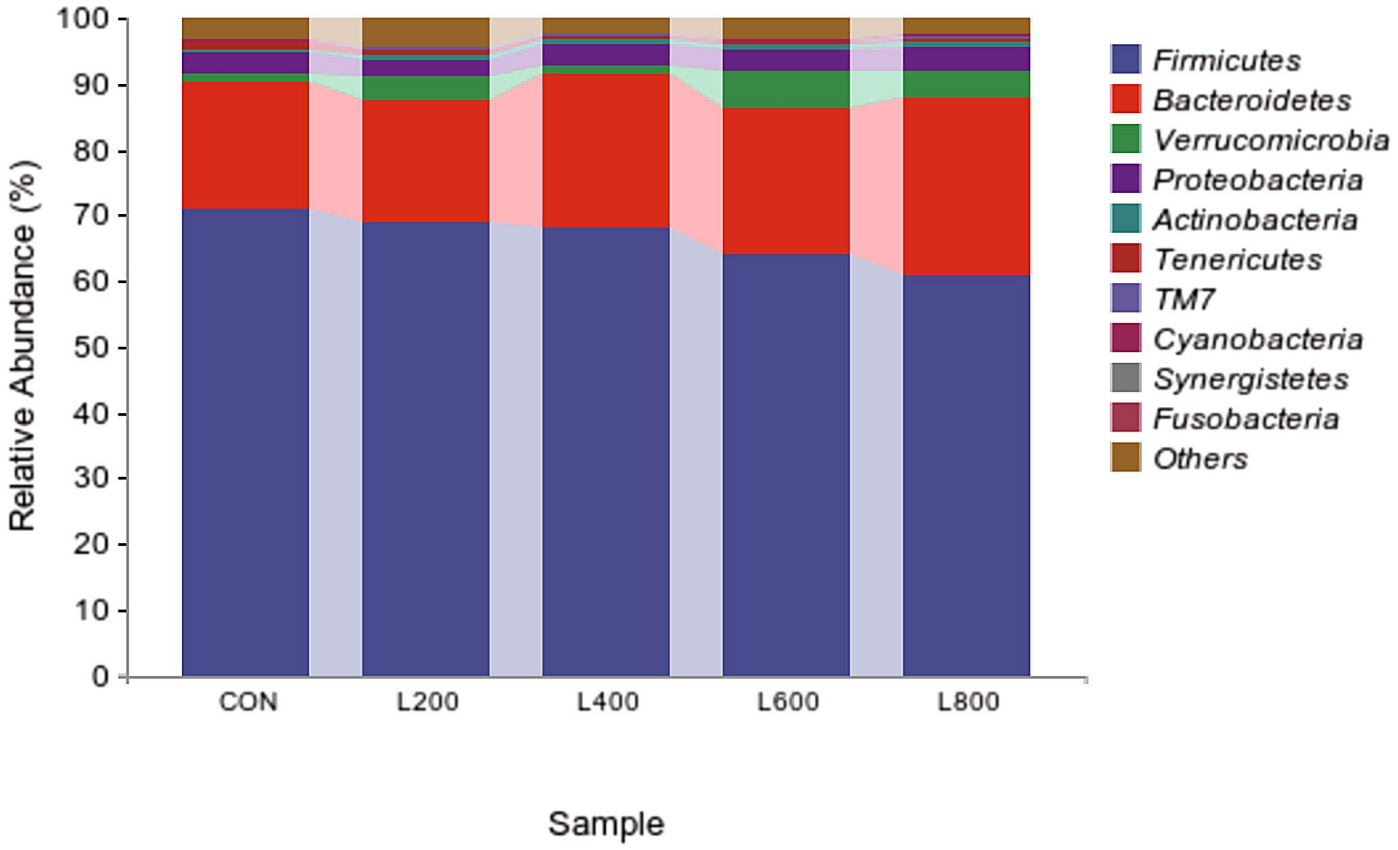
Relative abundance of cecum microbiota at phylum level.

**Figure 9:**
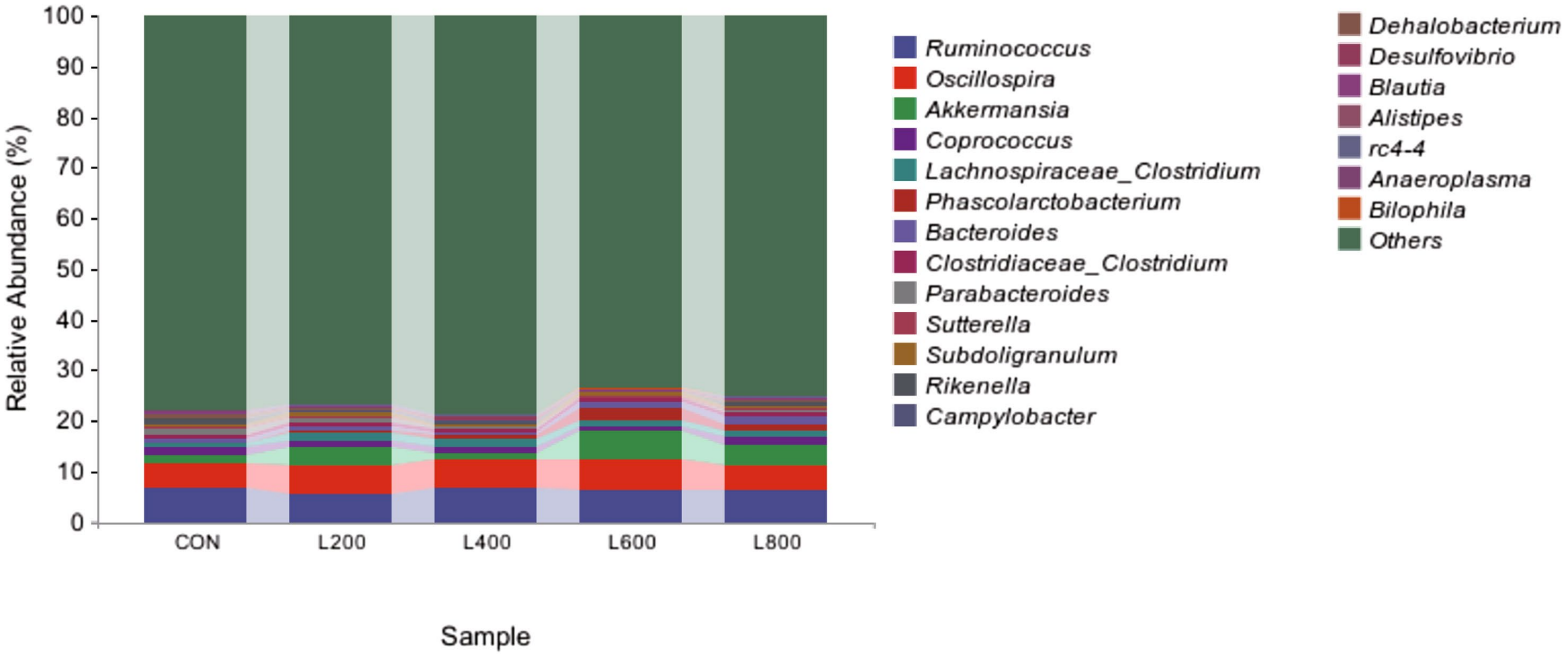
Relative abundance of cecum microbiota at genus level.

The operational taxonomic unit (OTU) analysis revealed varying numbers of identified OTUs across the experimental groups, with 4,605, 4,085, 4,421, 3,890, and 4,446 OTUs in the CON, L_200_, L_400_, L_600_, and L_800_ groups, respectively. Notably, 1,116 OTUs were shared among the four groups, as illustrated in Figure 5. The microbial diversity within an individual sample was assessed by the Chao1, Goods_coverage, Shannon, Simpson indices, and Observed_species but no significant differences between all groups were observed (Figure 6). Principal component analysis of OTUs was performed to evaluate the similarities and differences between the control group and the experimental group (Figure 7), and the results showed that the cecal microbiota of CON group was significantly separated from that of L_400_, L_600_, and L_800_ groups. Taxonomic profiling indicated that *Firmicutes* and *Bacteroidetes* accounted for most of the intestinal bacteria of rabbits (Figure 8). At the phylum level, there were no significant differences in the relative bacterial abundance among all groups (*p* > 0.05) (Table 4). The relative abundance of the 20 dominant genera in each group at the genus level was also analyzed. *Ruminococcus* and *Oscillospira* were found to be the main genera in the 4 treatment groups (Figure 9). As shown in Table 5, compared with the CON group, the relative abundance of *Phascolarctobacterium* in L_600_ and L_800_ groups was significantly increased, *Subdoligranulum* in L_200_ group was significantly increased, and *Dehalobacterium* in L_400_, L_600_, and L_800_ groups was significantly decreased. The relative abundance of *Alistipes* in group L_800_ was significantly increased (*p* < 0.05).

**Table 4:** Relative abundance of caecal microbiota at phylum level.

Item	Groups					SEM	*p*-value
	CON	L_200_	L_400_	L_600_	L_800_		
Firmicutes	70.81	68.79	68.25	64.08	60.97	1.668	0.360
Bacteroidetes	19.30	18.66	23.23	22.19	26.75	1.714	0.621
Verrucomicrobia	1.48	3.63	1.30	5.69	4.12	0.651	0.153
Proteobacteria	3.06	2.42	3.19	3.00	3.68	0.174	0.265
Actinobacteria	0.65	0.80	0.71	0.90	0.77	0.061	0.790
Tenericutes	0.88	0.82	0.57	0.51	0.65	0.078	0.540
TM7	0.24	0.27	0.16	0.19	0.30	0.032	0.647
Cyanobacteria	0.32	0.13	0.26	0.16	0.13	0.034	0.321
Firmicutes	0.00	0.13	0.04	0.02	0.06	0.029	0.710
Bacteroidetes	0.00	0.00	0.00	0.00	0.01	0.001	0.324

SEM, total standard error of the means (*n* = 4). ^a,b,c^Means with different superscripts in the same row are significantly different (*p* < 0.05).

**Table 5:** Relative abundance of caecal microbiota at genus level.

Item	Groups					SEM	*p*-value
	CON	L_200_	L_400_	L_600_	L_800_		
Ruminococcus	6.78	5.51	6.76	6.38	6.39	0.316	0.756
Oscillospira	4.90	5.42	5.55	5.90	4.67	0.319	0.788
Akkermansia	1.48	3.63	1.30	5.68	4.12	0.650	0.154
Coprococcus	1.43	1.47	1.28	0.72	1.68	0.200	0.668
Lachnospiraceae-Clostridium	1.16	1.50	1.33	1.22	1.10	0.103	0.798
Phascolarctobacterium	0.00^c^	0.32^c^	0.88^bc^	2.47^a^	1.43^b^	0.235	<0.001
Bacteroides	0.44	0.79	0.50	1.24	1.55	0.177	0.204
Clostridiaceae-Clostridium	1.10	0.78	0.88	0.66	0.66	0.057	0.066
Parabacteroides	0.99	0.95	0.23	0.24	0.36	0.206	0.640
Sutterella	0.61	0.59	0.23	0.49	0.55	0.080	0.618
Subdoligranulum	0.45^bc^	0.71^a^	0.25^c^	0.52^ab^	0.32^bc^	0.048	0.004
Rikenella	0.97	0.07	0.52	0.03	0.62	0.160	0.315
Campylobacter	0.22	0.18	0.42	0.06	0.32	0.061	0.442
Dehalobacterium	0.54^a^	0.40^ab^	0.05^c^	0.01^c^	0.18^bc^	0.061	0.008
Desulfovibrio	0.24	0.29	0.45	0.09	0.10	0.074	0.567
Blautia	0.19	0.24	0.18	0.16	0.23	0.015	0.437
Alistipes	0.06^b^	0.10^b^	0.12^b^	0.11^b^	0.32^a^	0.023	<0.001
rc4-4	0.11	0.16	0.12	0.10	0.10	0.011	0.610
Anaeroplasma	0.18	0.14	0.05	0.09	0.14	0.039	0.901
Bilophila	0.10	0.10	0.12	0.12	0.08	0.020	0.966

SEM, total standard error of the means (*n* = 4). ^a,b,c^Means with different superscripts in the same row are significantly different (*p* < 0.05).

### 3.8 Association of cecum microflora with immunity and antioxidant capacity

**Figure 10:**
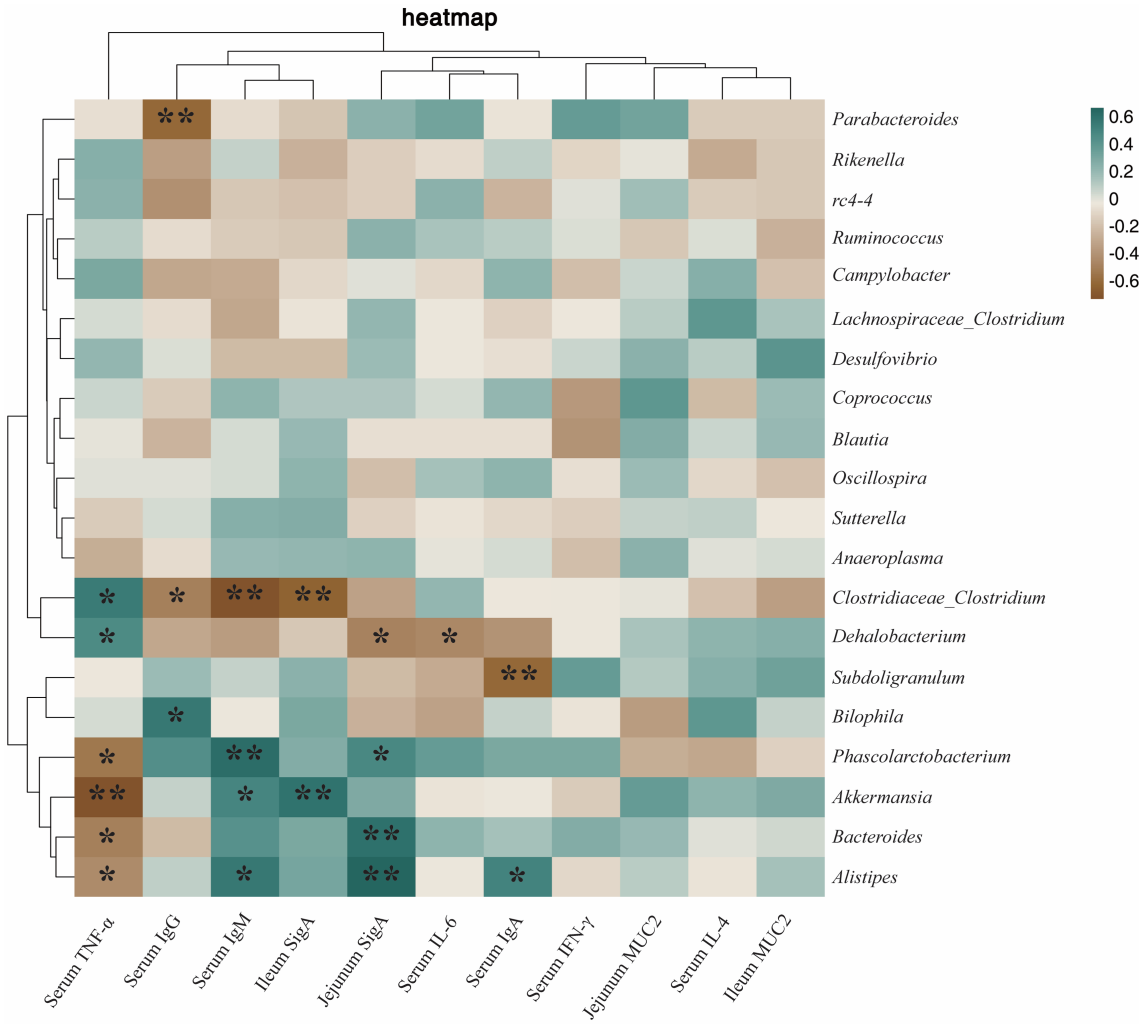
Heatmap of the correlation between cecum microbiota and immunity. The correlations were significant (*p < 0.05 and **p < 0.01), positive in green and negative in brown.

In order to further understand the potential relationship between gut microbes and immunity and antioxidant capacity, Spearman correlation analysis was used to analyze the relationship. As shown in Figure 10, *Phascolarctobacterium* was positively correlated with jejumum SIgA (*p* < 0.05) and serum IgM (*p* < 0.01), and negatively correlated with serum TNF-α level (*p* < 0.05). Serum IgA (*p* < 0.05), serum IgM (*p* < 0.05), and jejunum SIgA (*p* < 0.01) were positively correlated with *Alistipes*, and serum TNF-α (*p* < 0.05) was negatively correlated with *Alistipes*. *Parabacteroides* was negatively correlated with serum IgG (*p* < 0.01). *Clostridiaceae_Clostridium* was positively correlated with serum TNF-α (*p* < 0.05), and negatively correlated with serum IgG (*p* < 0.05), serum IgM (*p* < 0.01), and ileum SIgA (*p* < 0.01). *Dehalobacterium* was positively correlated with serum TNF-α (*p* < 0.05) and negatively correlated with serum IL-6 (*p* < 0.05), and jejunum SIgA (*p* < 0.05). *Subdoligranulum* was negatively correlated with serum IgA (*p* < 0.01). *Bilophila* was positively correlated with serum IgG (*p* < 0.05). *Akkermansia* was negatively correlated with serum TNF-α (*p* < 0.01), and positively correlated with serum IgM (*p* < 0.05), and ileum SIgA (*p* < 0.01). *Bacteroides* was negatively correlated with serum TNF-α (*p* < 0.05) and positively correlated with jejunum SIgA (*p* < 0.01).

**Figure 11:**
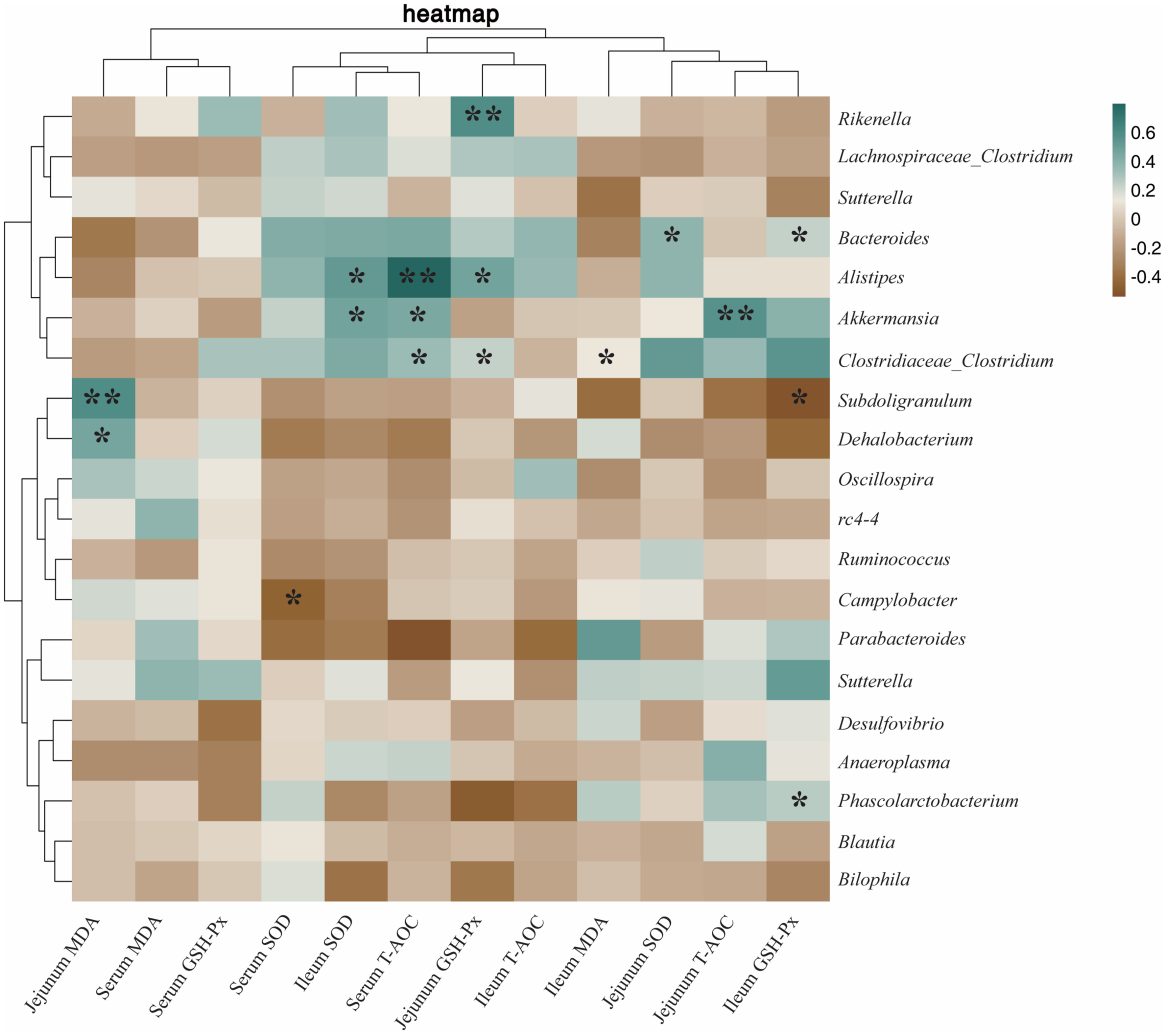
Heatmap of the correlation between cecum microbiota and antioxidant capacity. The correlations were significant (*p < 0.05 and **p < 0.01), positive in green and negative in brown.

Microorganisms associated with antioxidant results as shown in Figure 11, *Phascolarctobacterium* was positively correlated with ileum GSH-Px (*p* < 0.05). *Alistipes* was positively correlated with jejunum GSH-Px (*p* < 0.05), ileum SOD (*p* < 0.05), and serum T-AOC (*p* < 0.01). *Rikenella* was positively correlated with jejunum GSH-Px (*p* < 0.01). *Bacteroides* was positively correlated with jejunum SOD (*p* < 0.05) and ileum GSH-Px (*p* < 0.05). *Akkermansia* was positively correlated with ileum SOD (*p* < 0.05), serum T-AOC (*p* < 0.05), and jejunum T-AOC (*p* < 0.01). *Clostridiaceae_Clostridium* was positively correlated with serum SOD (*p* < 0.05) and jejunum GSH-Px (*p* < 0.05), and negatively correlated with ileum MDA (*p* < 0.05). *Subdoligranulum* was positively correlated with jejunum MDA (*p* < 0.01) and negatively correlated with ileum GSH-Px (*p* < 0.05). *Campylobacter* was negatively correlated with serum SOD (*p* < 0.05).

## 4 Discussion

The weaning stage poses a critical period for rabbits, susceptible to external stimuli such as dietary changes and environmental shifts, potentially leading to appetite loss, and even an increase in mortality and diarrhea rates (19). Zhu et al. (20) observed that the supplementation of 500 mg/kg of inactivated complex probiotics in the diet positively influenced cecum microbiota, consequently improving diet conversion and growth performance in yellow-finned broilers. Noteworthy findings by Tartrakoon et al. (21) demonstrated enhanced feed efficiency and ADG in pigs through the utilization of 20 mg/kg of heat-killed *L. plantarum*. Consistent with prior studies, the current research underscores that the addition of 600 mg/kg and 800 mg/kg of heat-killed LA significantly reduces F/G and enhances the growth performance of rabbits. This improvement in feed efficiency correlates with the observed elevation in the activity of digestive enzymes (lipase, protease, cellulase) within the intestinal tract of rabbits (22). Notably, the potential role of heat-killed LA in regulating gut microflora and diminishing the incidence of diarrhea aligns with the findings of Moal et al., who observed a reduction in harmful bacterial adhesion in the intestine through the use of inactivated probiotics (23), resulting in a decreased frequency of diarrhea in piglets (24). In summary, we hypothesize that the improved growth performance may also be related to heat-killed LA by modulating gut microflora and mitigating the occurrence of diarrhea.

Apparent digestibility serves as a key indicator of an animal’s proficiency in nutrient digestion within the diet, and this is intricately linked to digestive enzyme activity, contributing positively to growth performance (25, 26). Mammalian food nutrients primarily undergo digestion orchestrated by various digestive enzymes, with a minor portion being subject to digestion by intestinal bacteria (27, 28). Palamidi et al. (29) reported an elevation in lipase activity and improved digestibility in broilers when fed heat-killed complex probiotics. Similarly, Dawood et al. (30) observed a significant increase in amylase, protease, and lipase activities following the inactivation of *L. plantarum*, leading to enhanced nutrient digestion. In concordance with these findings, the current study demonstrated a substantial increase in trypsin, lipase, and cellulase activities upon supplementing diets with 600 mg/kg and 800 mg/kg of heat-killed LA. This trend aligned with the concurrent increase in the apparent digestibility of CP and CF in rabbits. Furthermore, the inactivation of LA was found to enhance the intestinal microbial composition, favoring an increased relative abundance of beneficial bacteria such as Lactobacillus and Bacillus in the intestine (31, 32). This augmentation in beneficial microbial communities within the gut can potentiate their ability to induce the production of endogenous enzymes (33, 34). Consequently, the inactivation of LA appears to contribute to the enhancement of digestive enzyme activity, ultimately improving digestibility and, consequently, the growth performance of rabbits.

Oxidation reactions, yielding ROS, are constant processes within organisms. Under normal physiological conditions, animal cells maintain a delicate equilibrium between ROS production and clearance (35). Excessive accumulation of free radicals poses a significant threat to tissues, leading to intestinal damage, nutrient malabsorption, and severe diarrhea (36, 37). Antioxidant defense enzymes, including SOD and GSH-Px, play pivotal roles in counteracting oxidative stress (38). SOD, functioning as the primary defense against free radicals, not only exhibits superb antioxidant effects but also inhibits the synthesis and release of inflammatory factors (39). T-AOC, encompassing both enzymatic and non-enzymatic antioxidant activities, provides a comprehensive assessment of the organism’s antioxidant status (40). Previous research, such as Liu et al. (41) and Dawood et al. (30) has highlighted the antioxidant properties of heat-killed LA and heat-killed *L. plantarum*, respectively. In this experiment, supplementation with heat-killed LA demonstrated a notable increase in SOD activity and T-AOC capacity in rabbits, with the most pronounced effects observed at supplementation doses of 600 mg/kg and 800 mg/kg. This augmentation in antioxidant-related enzyme activity may be attributed to the ability of heat-killed Lactobacillus to activate the Nrf2 signaling pathway (42, 43). Activation of the Nrf2 signaling pathway triggers the transcription and translation of downstream antioxidant genes (such as SOD and T-AOC), bolstering antioxidant status in response to environmental stress and sustaining normal intestinal function (44).

Immunoglobulin (IgA, IgM, and SigA etc.) is one of the components of animal immune system and an important parameter reflecting the immune capacity of animal body (45). IgA plays a crucial role in protecting the mucosal surface by preventing the entry, binding, and colonization of toxins and pathogens (46). IgM is the immunoglobulin with the strongest initial immune effect, and is an important barrier against pathogenic invasion (47). In this study, supplementation with heat-killed LA significantly increased the levels of IgA and IgM in serum, which reflected that heat-killed LA could improve the immune capacity of rabbits. As the main immune defense line of intestinal mucosa, SigA has the function of neutralizing pathogens and bacterial exotoxins in intestinal mucosa and maintaining the stability of intestinal flora (48). Danladi et al. found that supplementation of feed with inactivated *Lactobacillus plantarum* significantly increased the intestinal SigA content of broiler chickens and improved immunity (49). Consistent with the results of previous studies, we found that feed supplementation with 600 mg/kg and 800 mg/kg of heat-killed LA significantly enhanced the secretion of intestinal SigA, protected the intestinal immune barrier, and reduced the occurrence of intestinal diseases. TNF-α is a pro-inflammatory cytokine produced mainly by macrophages and monocytes, which is involved in the inflammatory response, and when its level is too high, it increases intestinal permeability and causes an enteritis response (50). It was found that LTA isolated from Lactobacillus can block the phosphorylation of NF-κB and the phosphorylation degradation of IkB, thereby inhibiting the expression of TNF-α (51). In this experiment, we found that supplementation with heat-killed LA reduced the serum levels of TNF-α. This may be related to the blocking of NF-κB phosphorylation by heat-killed LA. It was also shown that heat inactivation of LA does not cause an inflammatory response in the meat rabbit organism and has no adverse effect on its health.

There are a large number of microorganisms in the intestinal tract of animals, and their species composition and activity play an important role in the immune and inflammatory responses of animals (52, 53). Therefore, the balance of intestinal flora is related to the health of animals. Under normal conditions, the composition of the intestinal flora is relatively stable, but at the same time it can be disturbed by external factors (54). In the current study, probiotic supplements or probiotic products were found to be one of the effective ways to maintain a healthy balance of gut microbiota (55–58). In addition, species-rich communities enhance intestinal microecological stability and reduce susceptibility to bacterial invasion and intestinal inflammation (59). In this study, dietary heat-killed LA can regulate the cecal microflora structure of rabbits and increase the relative abundance of *Phascolarctobacterium* and *Alistipes*. *Phascolarctobacterium* and *Alistipes* are probiotics that promote intestinal health. Their main function is to produce short chain fatty acid (SCFAs), which play a vital role in maintaining nutrient metabolism and microbial homeostasis (60, 61). Acetic acid is not only the most abundant SCFAs in the gut, but also inhibits the NF-κB signaling pathway by down-regulating the expression of TLR gene, thereby reducing the content of TNF-α in the body and reducing the inflammatory response (62). In addition, SCFAs can also accelerate the activation, differentiation and antibody production of B cells by promoting the synthesis of acetyl-CoA, 5‘-adenosine triphosphate and fatty acids in B cells, thus promoting the secretion of SigA, IgA, and IgM (63, 64). SCFAs can also act as an activator of Keap1-Nrf2 signaling pathway (65, 66), and the activation of Nrf2 is positively correlated with SOD, GSH-Px, and T-AOC (44, 67). The above results showed that heat-killed LA can promote the proliferation of *Phascolarctobacterium* and *Alistipes*, thereby reducing serum TNF-α level, and increasing jejunum SigA, serum IgM, serum IgA, ileum GSH-Px, jejunum GSH-Px, ileum SOD and serum T-AOC levels, which may be due to the regulation of SCFAs by *Phascolarctobacterium* and *Alistipes*. These results provide a theoretical basis for understanding heat-killed LA-mediated microbial promotion of immunity and antioxidant capacity in rabbits. However, further microbiome-based studies are needed to elucidate the mechanisms by which *Phascolarctobacterium* and *Alistipes* promote immunity and antioxidant capacity in rabbits.

## 5 Conclusion

In conclusion, the incorporation of heat-killed LA into the diet demonstrates a capacity to enhance intestinal digestive enzyme activity, bolster antioxidant status, modulate the immune capacity, increase the relative abundance of beneficial bacteria in the cecum and foster overall organismal health in rabbits. This ultimately translates into improved growth performance. Upon comprehensive analysis, the recommended additive levels of heat-killed LA in rabbit diets appear to be 600 and 800 mg/kg.

## Data availability statement

The original contributions presented in the study are included in the article/supplementary material, further inquiries can be directed to the corresponding authors.

## Ethics statement

The animal study was approved by Animal Care and Use Committee of Hebei Agriculture University (Baoding, China). The study was conducted in accordance with the local legislation and institutional requirements.

## Author contributions

MX: Writing – original draft, Conceptualization, Funding acquisition, Project administration, Resources, Supervision, Validation, Writing – review & editing. CL: Writing – original draft, Conceptualization, Data curation, Formal analysis, Methodology, Writing – review & editing. DW: Writing – original draft, Data curation, Formal analysis, Investigation, Methodology, Resources, Software. FW: Methodology, Project administration, Software, Writing – review & editing. LK: Writing – original draft, Data curation, Formal analysis, Investigation, Methodology. ZJ: Writing – original draft, Data curation, Formal analysis, Investigation, Methodology. WH: Writing – original draft, Data curation, Formal analysis, Investigation, Methodology. SC: Writing – original draft, Data curation, Formal analysis, Investigation, Methodology. WF: Writing – original draft, Data curation, Formal analysis, Investigation, Methodology. YL: Writing – original draft, Conceptualization, Project administration, Resources, Supervision, Validation, Visualization, Writing – review & editing. BC: Writing – original draft, Funding acquisition, Supervision.
